# Dual effect of DLBCL-derived EXOs in lymphoma to improve DC vaccine efficacy in vitro while favor tumorgenesis in vivo

**DOI:** 10.1186/s13046-018-0863-7

**Published:** 2018-08-13

**Authors:** Zhenzhen Chen, Liangshun You, Lei Wang, Xianbo Huang, Hui Liu, Ju ying Wei, Li Zhu, Wenbin Qian

**Affiliations:** 1 Department of Hematology, the First Affiliated Hospital, College of Medicine, Zhejiang University, Zhejiang, 310003 Hangzhou China; 2 Malignant Lymphoma Diagnosis and Therapy Center, the First Affiliated Hospital, College of Medicine, Zhejiang University, Zhejiang, 310003 Hangzhou China; 3 Institute of Hematology, The First Affiliated Hospital, College of Medicine, Zhejiang University, 79# Qingchun Road, Hangzhou, 310003 People’s Republic of China

**Keywords:** Exosome, Diffuse large B-cell lymphoma, Dendritic cells, Cancer immunotherapy, Immunomodulation, Invasion, Tumor microenvironment

## Abstract

**Background:**

Exosomes derived from tumor cells (TEXs) are involved in both immune suppression, angiogenesis, metastasis and anticancer stimulatory, but the biological characteristics and role of diffuse large B cell lymphoma (DLBCL)-derived exosomes have been less investigated.

**Methods:**

Exosomes (EXOs) were isolated from OCI-LY3, SU-DHL-16, and Raji cells and biological characteristics of EXOs were investigated using electron microscopy, flow cytometry analysis, and Western blot analysis. The protein expression of EXOs was determined by an antibody array. Next, the communication between EXOs and lymphoma cell, stromal cell, dendritic cells (DCs), and T cells was evaluated. Finally, effect of DLBCL TEXs on tumor growth in vivo was investigated.

**Results:**

We demonstrated that EXOs derived from DLBCL cell lines displayed malignancy molecules such as c-Myc, Bcl-2, Mcl-1, CD19, and CD20. There was a different protein expression pattern between DLBCL TEXs and Burkitt lymphoma TEXs. DLBCL TEXs were easily captured by DCs and lymphoma cells, and mainly acted as an immunosuppressive mediator, evidenced by induction of apoptosis and upregulation of PD-1 in T cells. Furthermore, the TEXs stimulated not only cell proliferation, migration of stromal cells but also angiogenesis. As a result, the TEXs promoted tumor growth in vivo. On other hand, DLBCL TEXs did not induce apoptosis of DCs. After pulsed with the TEXs, DCs could stimulate clonal expansion of T cells, increase the secretion of IL-6 and TNFα, and decrease the production of immunosuppressive cytokine IL-4 and IL-10. The T cells from tumor bearing mice immunized by TEX were shown to possess superior antilymphoma potency relative to immunization of tumor lysates.

**Conclusions:**

This study provides the framework for novel immunotherapies targeting TEXs in DLBCL.

**Electronic supplementary material:**

The online version of this article (10.1186/s13046-018-0863-7) contains supplementary material, which is available to authorized users.

## Background

Diffuse large B-cell lymphoma (DLBCL), a clinically and biologically heterogeneous tumor, is the most common subtype of lymphoma, representing of 20–30% of all lymphoproliferative disorders [1]. Although 50–70% of DLBCL patients can be cured by current standard treatment with rituximab-based chemotherapy, about 50% of the patients are found to be inadequate by this treatment, in which 20% of patients suffer from primary refractory and others relapse after achieving complete remission [2]. Most patients with refractory DLBCL have no effective treatment options. In the last years, immunotherapy, an alternative method, appears promising and probably will improve therapeutic strategy for the patients with DLBCL. Immunotherapies fall into four categories such as immune-checkpoint inhibitors, adoptive cellular therapy including chimeric antigen receptor T-cell, and therapeutic cancer vaccines [3–5]. Among them, dendritic cell (DC)-based vaccines offer a promising therapeutic platform for a variety of cancer including lymphoma. For example, a pilot study demonstrated that vaccination with DCs loaded with apoptotic and necrotic autologous tumor cells increased natural killer (NK) cell activation, reduced Treg frequency and induced both T- and B-cell anticancer responses associated with clinical efficacy in heavily pretreated B cell lymphoma patients [6].

Exosomes (EXOs) are membrane vesicles with a diameter of 30–100 nm originating from multivesicular bodies of many types of cells including cancer cells, which function as a mode of intercellular communication and molecular transfer [7, 8]. Recently, tumor-derived exosomes (TEXs) have been shown in various cancer models to actively promote tumorigenesis and metastasis through intricate mechanisms including transfer of oncogenic receptors, protein and RNA, suppression of the function of NK cells and T cells, promotion of T regulatory cell expansion, and mediation of tumor microenvironment (TME) via angiogenesis promotion, stromal remodeling, and signaling pathway activation [9–14]. Although TEXs are predominantly immunosuppressive, they can also enhance immunostimulation and therefore serve as cancer vaccines. TEXs bear major histocompatibility complex (MHC) protein, chaperones, such as heat shock protein-70 (HSP-70) and/or HSP-90, and tumor-associated antigens (TAAs) taken up by DCs, which are effective in mediating anti-tumor immunity in vitro and in vivo [7, 15, 16]. The anti-cancer efficacy of TEXs was also confirmed in lymphoma. Menay*et al* [17] demonstrated that T cells from T-cell lymphoma TEXs-immunized mice secrete interferon-γ in response to tumor stimulation and administration of the TEXs into mice induces a tumor-specific immune response. In a mouse model, EXOs obtained heat-shocked B lymphoma cells (HS-Exo) had been shown to contain HSP-60, HSP-90 and molecules involved in immunogenicity including MHC class I, MHC class II, CD40 and CD86, and to induce maturation of DCs. Furthermore, HS-Exo immunization strong activated T cell response [18]. The dual role of EXOs from B-cell lymphoma already has been characterized extensively; however, there are only a few studies [19–21] that elucidate characteristic of the EXOs secreted by DLBCL cells.

In this study, we report a comprehensive analysis of EXOs derived from DLBCL cell lines and their role in the communication with T cells, DCs, and stromal cells including human umbilical vein endothelial cells (HUVEC) and human fibroblasts. More specifically, our results suggest a novel strategy by targeting TEXs in lymphoma therapeutic development.

## Methods

### Cell lines

The human DLBCL cell lines OCI-LY3 and SU-DHL-16 were kindly provided by Professor Jianyou Gu, Zhejiang Provincial Hospital of TCM (Hangzhou, China). The human Burkitt lymphoma cells Raji, HUVEC and the murine B lymphocyte cell line A20 were purchased from the American Type Culture Collection (ATCC; Manassas, VA, USA). The human dendritic cell line DCS, the normal human T cell line Th2, the human skin fibroblasts HSF and the murine DC cell line D2SC/1 were purchased from Huazhong University of Science and Technology (Wuhan, China). The SU-DHL-16, Raji and A20 cells were cultured in RPMI 1640 medium supplemented with 10% depleted fetal bovine serum (FBS; Gibico, Grand Island, NY, USA), which obtained by ultracentrifugation at 100,000 *g* for 18 h to remove possible FBS-containing EXOs. The OCI-LY3 cells were cultured in Iscove’s Modified Dulbecco’s Medium with 15% FBS. The DCS, Th2, HUVEC, HSF and D2SC/1 cells were cultured in Dulbecco’s Modified Eagle’s Medium with 10% FBS.

### Human DCs and fibroblasts (SFs)

Human peripheral blood mononuclear cells (PBMNCs) were obtained from healthy volunteers (provided by Zhejiang Blood Center, Zhejiang, China), and SFs were isolated from non-tumoral gastric walls of the patients who underwent surgery in our hospital. Informed consent was obtained from all volunteers and patients. PBMNCs were isolated using a human Lymphoprep solution (Axis-shield PoC AS, Oslo, Norway), and cultured in a 10-cm Petri dish and incubated for 24 h to allow them to adhere to the dish’s surface. Adherent cells were induced to form immature DCs, supplemented with 120 ng/mL recombinant human granulocyte macrophage colony-stimulating factor (PeproTech, Offenbach, Germany), and 60 ng/mL recombinant human interleukin-4 (PeproTech) to decrease contamination by macrophages for 5 days. Nonadherent cells were harvested and used as human lymphocytes. SFs were prepared by transferring the gastric tissue to a T25 flask and cutting into 1mm^3^ pieces. Incubate the chopped material with 5 ml of trypsin EDTA for 5 min at 37 °C, and then the trypsin was Inactivated by adding 1 ml FBS. The cell pellet was obtained by centrifuging of suspension at 200 *g* speed for 10 min, which was transferred to a T25 flask containing DMEM medium with 20% FBS and 2 × Penicillin/Streptomycin) after resuspend using DMEM.

### Murine bone marrow stromal cells (BMSCs)

The BMSCs were isolated from the bone marrow of C57BL/6 mice. The medullar canal of the tibias and femurs was flushed with PBS. The resulting suspension was harvested and filtered through a 70 μm cell strainer. After centrifugation, BMSCs were resuspended in red blood cell lysis buffer to remove red blood cells.

### Preparation of exosomes and cell lysates

The supernatant was sequentially centrifuged at 500 *g* for 10 min, followed by 2000 *g* for 30 min, and filtered with a 0.22 μm filter (Millipore, Bedford, MA, USA) to remove cells and cellular debris. The EXOs were isolated by ultracentrifugation at 110,000 *g* for 70 min at 4 °C. EXOs pellets were washed with phosphate-buffered saline (PBS) and ultracentrifugated at 110,000 *g* for 70 min. The cell lysates were obtained by five successive cycles of freeze-thawing. Cell lysates were then followed by centrifugation at 3000 *g* for 30 min, and filtered with a 0.22 μm filter. The protein concentration of EXOs and cell lysates were quantified by the Bradford assay (Sangon Biotech, shanghai, China).

### Nanoparticle tracking analysis (NTA)

The size distributions and surface Zeta potential of DLBCL TEXs were analyzed by NanoSight NS300 (NanoSight, Amesbury, United Kingdom) using NTA 3.2 software, as described elsewhere [22–24]. EXOs were measured upon dilution into PBS at a concentration of 6 × 10^8^ particles/ml in triplicates.

### Phenotypic characterization of exosomes

A total of 30 μg EXOs were precoated with 10 μL aldehyde/sulfate latex beads (Invitrogen, Carlsbad, CA, USA) overnight at room temperature and stopped by 0.1%BSA. EXOs, coated on Beads were stained with a panel of fluorescein APC-or PE-conjugated antibodies, CD19, CD20, CD40, CD80, CD83, CD86 and HLA-DR (BioLegend, San Diego, CA, USA) and the corresponding isotype-matched antibodies, and then followed by flow cytometry analysis (FACS; Accuri C6, BD, Franklin Lakes, NJ, USA).

### Transmission electron microscopy (TEM)

EXOs were loaded onto the shiny side of the copper grid and stained with 2% uranyl acetate for 3 min at room temperature (RT). Incubate the grid on top of some small drops of ultrapure water 2 min for each wash. Blotting the grid at 45-degree angle once from the side of the grid to remove excess solution by filter paper. The grid was observed with TEM (JEM-1200EX, JEOL, Japan).

### T cell proliferation assay

T lymphocytes derived from human PBMCs were stained with carboxy fluorescein diacetatesuccinimidyl ester (CFSE; Life Technologies, Waltham, MA, USA). DCs were pretreated with 10 μg/mL mitomycin C (Sigma, St Louis, MO, USA) to inhibit cells division. CFSE-labeled T cells were cocultured with DCs, DClys and DCtex at different ratios for 4 days. Subsequently, T cells were counterstain with anti-CD3-APC (BioLegend) for 30 min, and then analyzed by FACS.

### Cytokine release assay

Cytokines including IL-2, IL-4, IL-6, IL-10, IFN-γ, and TNF-α in supernatants of T lymphocytes co-cultured with DCs in vitro were detected using Cytometric Bead Array (CBA) Human Th1/Th2 Cytokine Kit (BD, Franklin Lakes, NJ, USA). The data were analyzed by the BD Biosciences CBA analysis software.

### Cytotoxicity assay

Cytotoxic lymphocyte activity was evaluated by a cytotoxicity detection kit (Promega, Madison, WI, USA), by measuring the cytolysis rate elicited by effector T lymphocytes against tumor cells. The indicator for cytotoxicity was the amount of lactate dehydrogenase released from lysed target cells. Red blood cell-depleted splenic lymphocytes and lymph nodes cells from the mice immunized with EXOs were harvested after 7 days of immunization as effector T lymphocytes. An MTT (3-(4,5-dimethylthiazol-2-yl) -2,5-diphenyltetrazolium bromide; Sigma) assay was used for evaluation of the effects of TEXs on proliferation of stromal cells as described previously [25]. Briefly, cells were seeded in 96-well plates at a density of 2 × 10^4^ cells/ml. After treated with different concentrations of TEXs for 96 h, the MTT assay was performed.

### Annexin V/PI binding assays

Cells were cultured at a density of 10^5^ cells/ml in a 6-well plate and treated with different concentrations of TEXs for 24 h. Apoptotic cells were quantified by propidium iodide (PI) and Annexin V-FITC double staining using a detection kit purchased from MultiSciences Biotech (Hangzhou, China) according to the manufacturer’s instructions, then analyzed by FACS analysis.

### Western blot analysis

Western blotting was performed as described previously [25]. EXOs and cell lysates were immunoblotted with these antibodies: anti-CD63, CD81, TSG101, Alix, Cytochrome C, Hsp70, MMP-2, MMP-9, CTLA-4, and c-Myc (Abcam, Cambridge, MA); anti-Bcl-2, Bcl-xl, Mcl-1, xIAP, TGF-β, TRAIL, GSK-3β, and FASL (Cell signaling technology, Beverly, MA); anti-FAS and BTLA (Proteintech, Rocky Hill, NJ, USA). The horseradish peroxidase-conjugated secondary antibody was obtained from MultiSciences Biotech, Hangzhou, China. Immunoblotting with anti-actin or anti GAPDH (Cell signaling technology) confirmed equivalent protein loading.

### Confocal laser scanning microscopy

Cells was incubated in complete medium with 30 μg PKH67(sigma)-labeled EXOs for 0–24 h at 37 °C, and then were washed twice with PBS, fixed in 4% formaldehyde (sigma) for 10 min at RT. After washed twice with PBS, co-stained with PE-anti CD19 for 30 min and 1 μM DAPI (Southern Biotech, Birmingham, AL, USA) for 5 min, cells were coated on microscopy slides and analyzed by a confocal microscope (Nikon C1-Si, Japan).

### Label-based human antibody array

The protein expressions of TEXs or cell lysates were analyzed by RayBio Biotin Label-based Human Antibody Array (Raybiotech, Norcross GA). This antibody array experiment was performed by Wayen Biotechnology (Shanghai, China) according to their established protocol. In brief, the samples were biotinylated and dialyzed, and then were added to the array and incubated overnight at 4 °C. After incubation with Cy3-Conjugated Streptavidin, the slides were scanned on a GenePix 4000 scanner and the images were analyzed with GenePix Pro 6.0 (Molecular Devices, Sunnyvale, CA).

### Quantitative real-time PCR

Total RNA was isolated using TRIzol reagent (Invitrogen), according to manufacturer’s instructions. cDNA was made from total RNA using reverse transcriptase kit (TaKaRa Shuzo, Kyoto, Japan), which was amplified on an Applied Biosystems 7500 real-time PCR system (Foster City, CA, USA). The specific primers (Invitrogen) for MMP-2 were 5′- CATTTGGCGGACTGT-3′ (forward) and 5′- AGGGTGCTGGCTGA -3′ (reverse), and for MMP-9 were 5′- TTGACAGCGACAAGAAGT-3′ (forward) and 5′- GGGCGAGGACCATAGA -3′ (reverse). The specific primers for GAPDH were 5’-GTCATCACCATTGGCAATGAG-3′ (forward) and 5’-CGTCACACTTCATGATGGAGTT-3′ (reverse).

### Transwell invasion assay

The effect of TEXs on cell invasion was determined by Transwell invasion assay as described previously [26]. Briefly, cells in DMEM were stimulated with EXOs (100 μg/mL) or vehicle (PBS) alone for 4 h at 37 °C, and then were collected and seeded onto the Matrigel-coated transwell inserts (Corning Inc., Corning, NY, USA). The inserts were placed onto a 24-well plate that contained DMEM (5% FBS). After incubated for 24 h at 37 °C to facilitate invasion, cells were fixed in 4% formaldehyde for 15 min and stained with 0.1% crystal violet for 20 min at RT. The invaded cells were imaged using light microscope and counted. Five fields of view were obtained per insert (*n* = 3 biological replicates).

### Matrixgel plug assay

Six-week-old NOD/SCID mice were injected subcutaneously along the abdominal midline with or withour 500 μL growth factor-reduced Matrigel containing OCI-LY3 EXOs (100 μg). Mice were sacrificed 14 days later, Matrigel plugs were removed, fixed in 4% formaldehyde, and embedded in paraffin. The paraffin sections were stained with hematoxylin/eosin (H & E) or stained with anti-CD31 (Abcam), followed by an Alexa Fluor 488-conjugated goat anti-mouse antibody (Invitrogen).

### Scratch wound assay (SWA)

SWA was performed as described previously [27]. Briefly, cells (2 × 10^4^) were incubated at 37 °C for 24 h. A scratch (wound) was performed on monolayer of cells along the vertical axis of each well under a light microscope. All the experiments were carried out in three replicates and three measurements were taken for each wound.

### Animal experiment

All animal experiments were carried out in the animal research center of Zhejiang Chinese Medical University (Zhejiang, China).C57BL/6 mice, BALB/C mice and NOD-SCID mice were purchased from Shanghai Slac Laboratory Animal CO. LTD (Shanghai, China). To examine whether EXOs could induce protective antitumor immunity, BALB/C mice were intravenously immunized with EXOs (10 μg/mouse). The mice, injected with PBS were considered as control. The tail blood samples were harvested for FACS after 7 days of immunization. To investigate immunomodulatory effect of EXOs in DC-vaccination, DCs activated by EXOs or tumor cell lysates were injected intravenously into MHC-matched BALB/C mice 3 times at weekly interval. The immunized mice were then challenged with 5 × 10^6^ tumor cells. In addition, for the mouse tumor model, six-week-old NOD-SCID mice were challenged subcutaneously in the flank with tumor cells, supplemented with 200 μg DLBCL EXOs. The tumors cells were injected subcutaneously with PBS as the control.

### Statistical analysis

Experimental results were analyzed by one-way analysis of variance. The *P*-value, below 0.05 was considered as statistically significant. All data are presented as mean ± standard deviation.

## Results

### The characterization of exosomes from B cell lymphoma

**Fig. 1:**
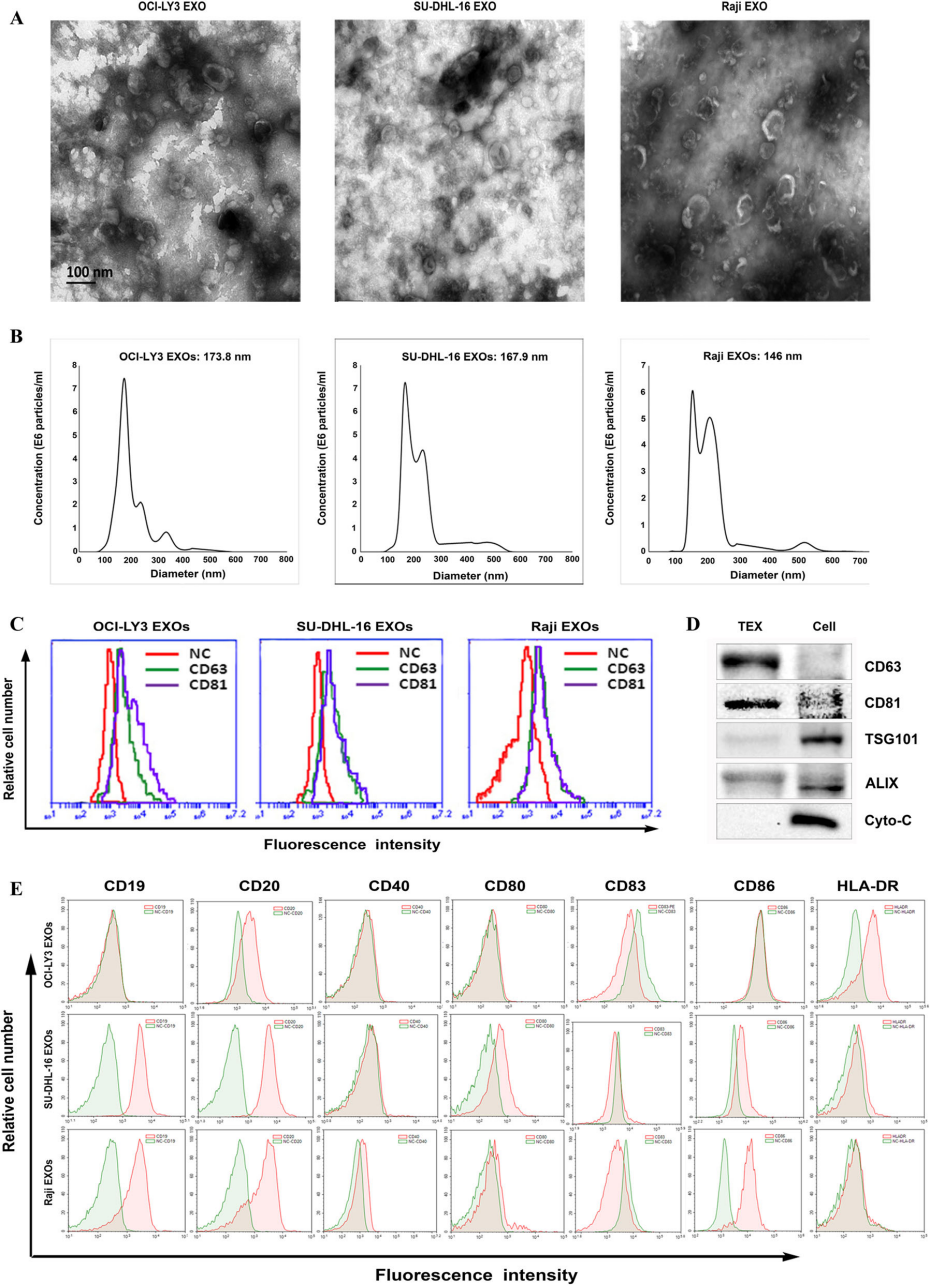
Characterization of exosomes derived from B cell lymphoma. **a** Transmission electron microscopy analysis of exosomes (EXOs) derived from DLBCL cell lines OCI-LY3, SU-DHL-16, and Burkitt’s lymphoma cell line Raji. (scale bar = 100 nm). **b** Mean diameter and size distribution of B cell-derived EXOs was assessed by Nanoparticle tracking analysis. **c** Flow cytometry analysis of the tetraspanins CD81 and CD63 on three kinds of lymphoma EXOs. **d** Western blotting analysis of exosomal biomarkers and cellular proteins in OCI-LY3 EXOs and cell lysates. **e** B cell-derived EXOs were incubated with 4-μm-diameter aldehyde/sulfate latex beads, and stained with a panel of antibodies, followed by flow cytometry analysis. The EXOs were also stained with isotype-matched irrelevant antibodies respectively, which were used as controls (green line). Representative of the three independent experiments are shown

To analyze the characterization of EXOs derived from DLBCL, OCI-LY3 (a non-germinal center B cell like [GCB] lymphoma cell line), SU-DHL-16 (a GCB subtype cell line), and Burkitt’s lymphoma cell line Raji were used, and morphology of the EXOs was examined by TEM analysis that showed a population with typical exosomal pellet (Fig. 1a). Nanoparticle tracking analysis showed a population of EXOs came from OCI-LY3, SU-DHL-16 and Raji cells, and that mean diameter of TEXs was 173.8 nm, 167.9 nm and 146 nm, respectively (Fig. 1b). These finding are consistent with recent observation that size distribution of EXOs-derived from chronic lymphocytic leukemia (CLL) was 70 to 200 nm [28]. Next, we determined the surface Zeta potential of TEXs by NTA analyses. Results showed that the mean surface Zeta potential of EXOs from OCI-LY3, SU-DHL-16 and Raji cells was − 9.11 mV, − 8.67 mV, and-12.71 mV, respectively.

### Phenotype characterization of TEXs of B-cell lymphoma

To investigate its marker, TEXs coated on beads were stained with a panel of FITC-labelled antibodies, and then followed by FACS analysis. There is high expression of exosomal marker proteins CD63 and CD81, with a range of 55.3% to 75.2% in beads (Fig. 1c). Western blotting analysis also showed that CD81 and CD63, as well as the endocytic pathway and formation associated proteins TSG-101 and Alix were expressed in OCI-LY3TEXs (Fig. 1d). Taken together, these data confirmed the presence of TEXs. We further compared the phenotype of OCI-LY3, SU-DHL-16, and Raji cells with their corresponding TEXs. EXOs of SU-DHL-16, and Raji cells expressed CD19 and CD20, whereas OCI-LY3 EXOs only expressed CD20 (Fig. 1e), which is consistent with the results of their parent cell lines (Additional file 1: Figure S1). In addition, all three cell lines showed positive expression of costimulatory molecules including CD40, CD80, CD83 and CD86 (Additional file 1: Figure S1); however, only HLA-DR expression was positive in OCI-LY3 EXOs, while expression of CD80, CD86, and HLA-DR was positive in SU-DHL-16TEXs (Fig. 1e). Interestingly, the expression of surface markers related to specific malignancy lineages, such as CD19 and CD20, and costimulatory molecules in DLBCL TEX was much weaker than that in their parent cell lines.

### DLBCL-derived exosomes harbor TAAs and are easily taken up by DCs and lymphoma cells

**Fig. 2:**
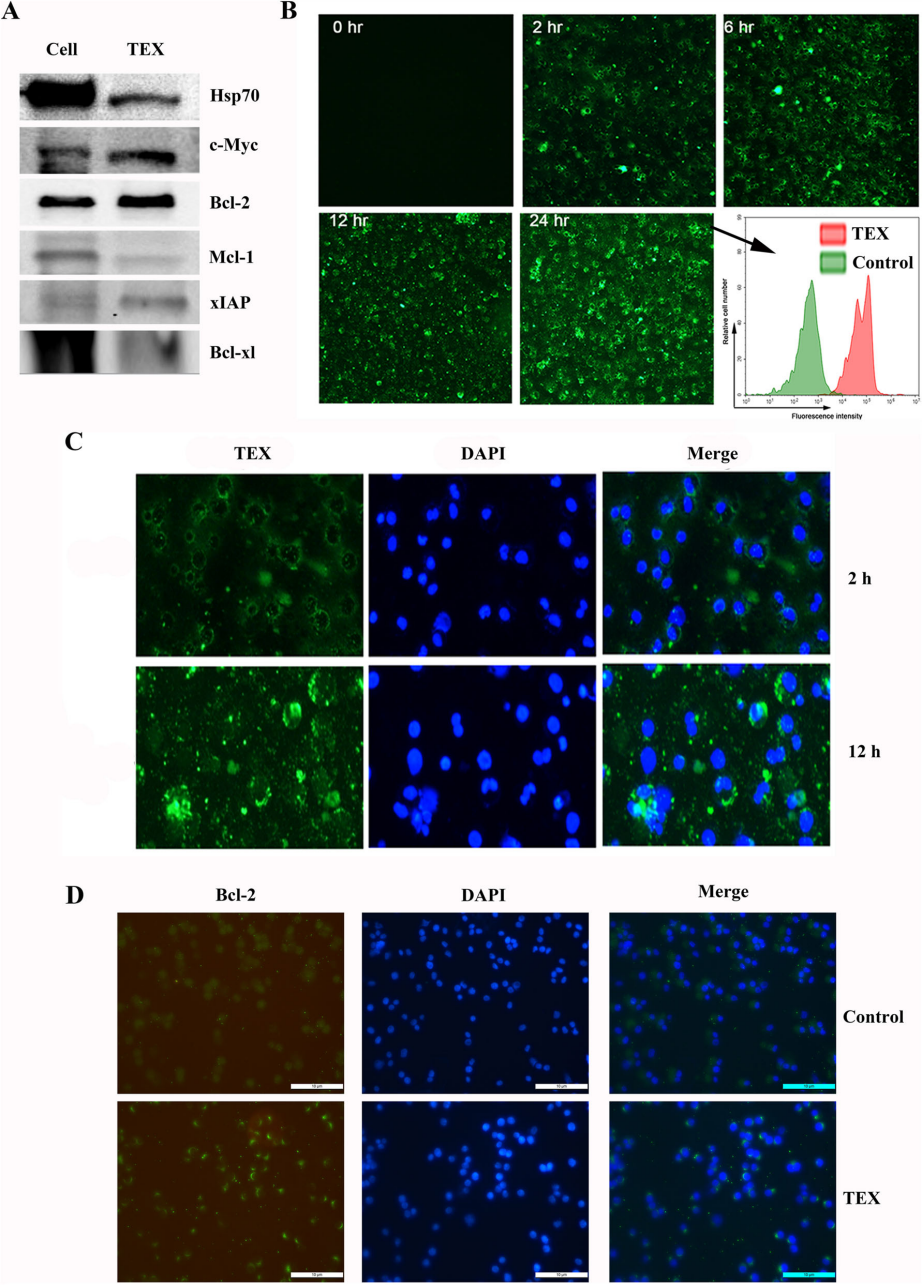
Exosomes derived from DLBCL cells harbor tumor associated antigens and internalized by DCs. **a** Immunoblot analysis of proteins from OCI-LY3 cells and its EXOs for detection of Hsp70, c-Myc, Bcl-2, Mcl-1, xIAP, and Bcl-xL. **b** DCs were cultured with PKH67 labeled OCI-LY3 EXOs (100 μg/ml) for the indicated times, and the uptake of EXOs was analyzed by immunofluorescence. Insert image represents the results of flow cytometry analysis for detection of PKH67 positive DCs at 24 h. **c** DCs were cultured with PKH67 labelled OCI-LY3 EXOs for 2 h and 12 h, respectively, and then counterstained with DAPI. Green represents PKH67-labelled EXOs; blue represents nuclei. **d** Human DC cell line DCS were treated with or without OCI-LY3 EXOs for 24 h, and then the expression of Bcl-2 were analyzed by immunofluorescence method

**Fig. 3:**
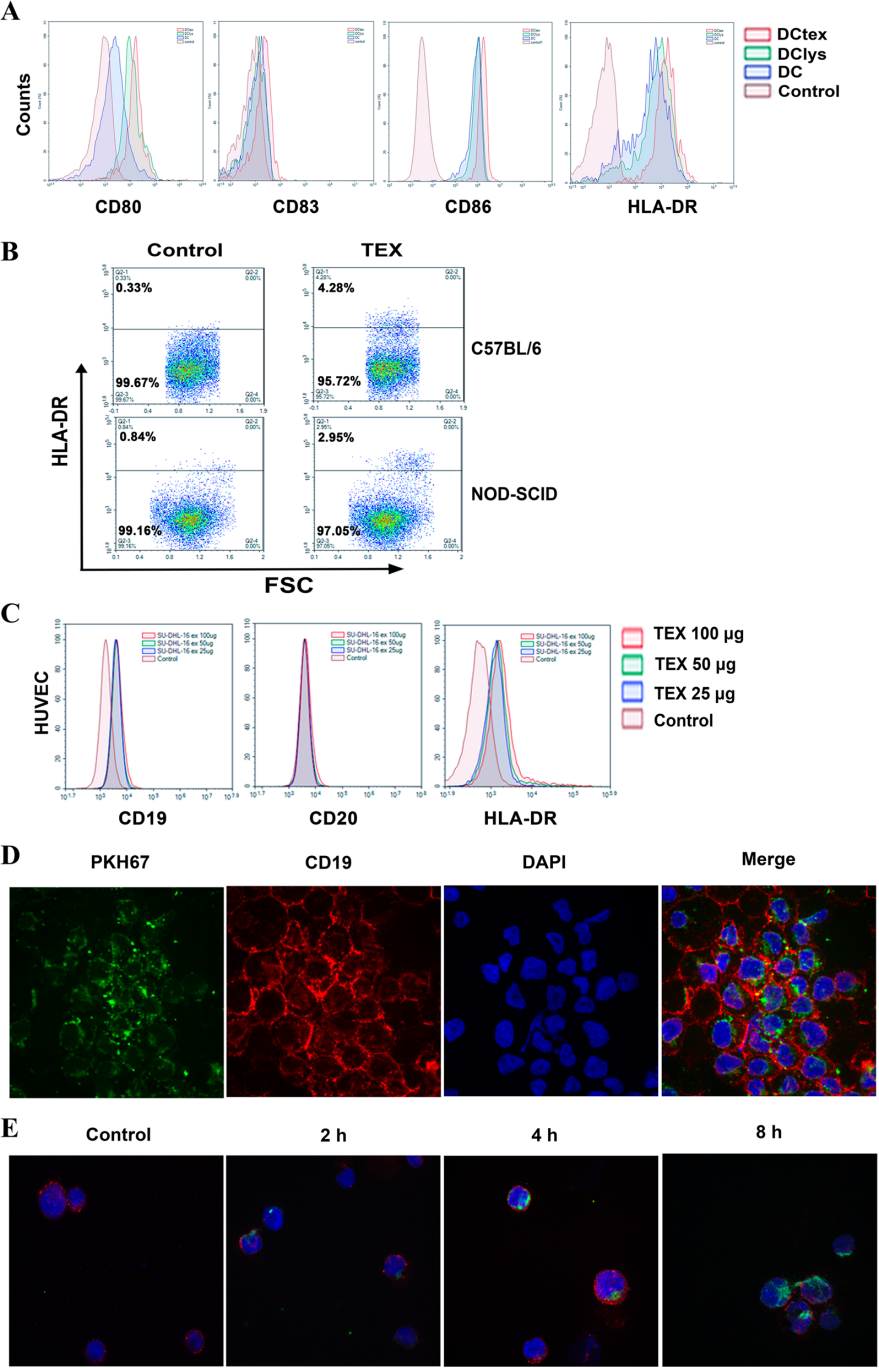
Communication between DLBCL exosomes and recipient cells. **a** Human DCs were treated with or without OCI-LY3 EXOs (50 μg/mL; DCtex), OCI-LY3 lysates (50 μg/mL; DClys), for 24 h, and stained with anti-CD80, CD83, CD86, and HLA-DR. Flow cytometry analysis was used for the detection of protein expression. **b** Murine bone marrow stromal cells (BMSC) obtained from C57BL/6 and NOD-SCID mice were incubated with or without OCI-LY3 EXOs (50 μg/mL) for 24 h. Human HLA-DR expression in the BMSC was assessed by flow cytometry analysis. **c** HUVECs were treated with or without different concentrations of SU-DHL-16 EXOs for 24 h. The expression of B cell markers CD19, CD20, and HLA-DR were analyzed by flow cytometry analysis. **d** and **e** PKH-67 labelled-EXOs derived from Raji cells were incubated with Raji cells for the indicated time, and then cells were stained with PE-anti CD19, followed by counterstained with DAPI. EXOs uptake and cellular location was assessed under a fluorescence microscopy

It is reported that TEXs contain a broad variety of proteins, lipids and glycans as well as native tumor-antigens that can be efficiently taken up by DCs [29, 30], and that TEXs produced by different tumor cells carry distinct molecular signatures [7]. Thus we examined the expression level of c-Myc, and Bcl-2 family proteins such as Mcl-1, Bcl-xl and xIAP, which provide aberrant survival advantage for lymphoma cells [31], in OCI-LY3 EXOs and its parental cell line (Fig. 2a). The all molecules were detected both in OCI-LY3 EXOs s and cell lysates of OCI-LY3, but the expression level of the proteins except c-Myc in the TEXs was lower than that of OCI-LY3 cells. The OCI-LY3 EXOs also contained less HSP-70 than cell lysates of OCI-LY3. Next, we evaluated whether DLBCL TEXs are taken up by DCs by treating DCs with OCI-LY3 EXOs that were stained with PKH67, a green membrane dye. Results showed that the cellular uptake of TEXs was readily detectable at 2 h and peaked at 24 h after the treatment (Fig. 2b), and that internalized PKH67-labeled TEXs were in perinuclear region of the cells (Fig. 2c). Using immunofluorescence method, we demonstrated the expression of Bcl-2 in human DC cell line DCS treated with OCI-LY3 EXOs (Fig. 2d). Interestingly, we found that treatment of DCs with TEXs resulted in DC maturation and activation as demonstrated by elevated levels of co-stimulatory molecules (CD80, CD83 and CD86) and human leukocyte antigen HLA-DR expression on DCtex surface compared with DClys and untreated DCs (Fig. 3a), suggesting OCI-LY3 EXOs is capable of activating DCs. We further investigated the ability of lymphoma TEXs to transfer their cargos into other kind of cells. As shown in Fig. 3b, HLA-DR expression was found in C57BL/6 and NOD-SCID mouse bone marrow cells co-cultured with OCI-LY3 EXOs, while both HLA-DR and CD19 expression were also observed in HUVECs treated with SU-DHL-16 EXOs (Fig. 3c).

In order to study the capture of TEXs by lymphoma cells, we treated Raji cells with the PKH-67 labeled Raji-derived EXOs. After incubation for 24 h, the cell membrane was stained with PE-anti CD19 antibody, and TEXs internalization was checked by confocal microscopy that showed apparent internalization (Fig. 3d). Time kinetics indicated TEXs accumulation in Raji cells in a time-dependent manner (Fig. 3e), consistent with published results of mantle cell lymphoma (MCL) [22].

### Lymphoma TEXs stimulate T cell function and proliferation via the host DCs in vitro

**Fig. 4:**
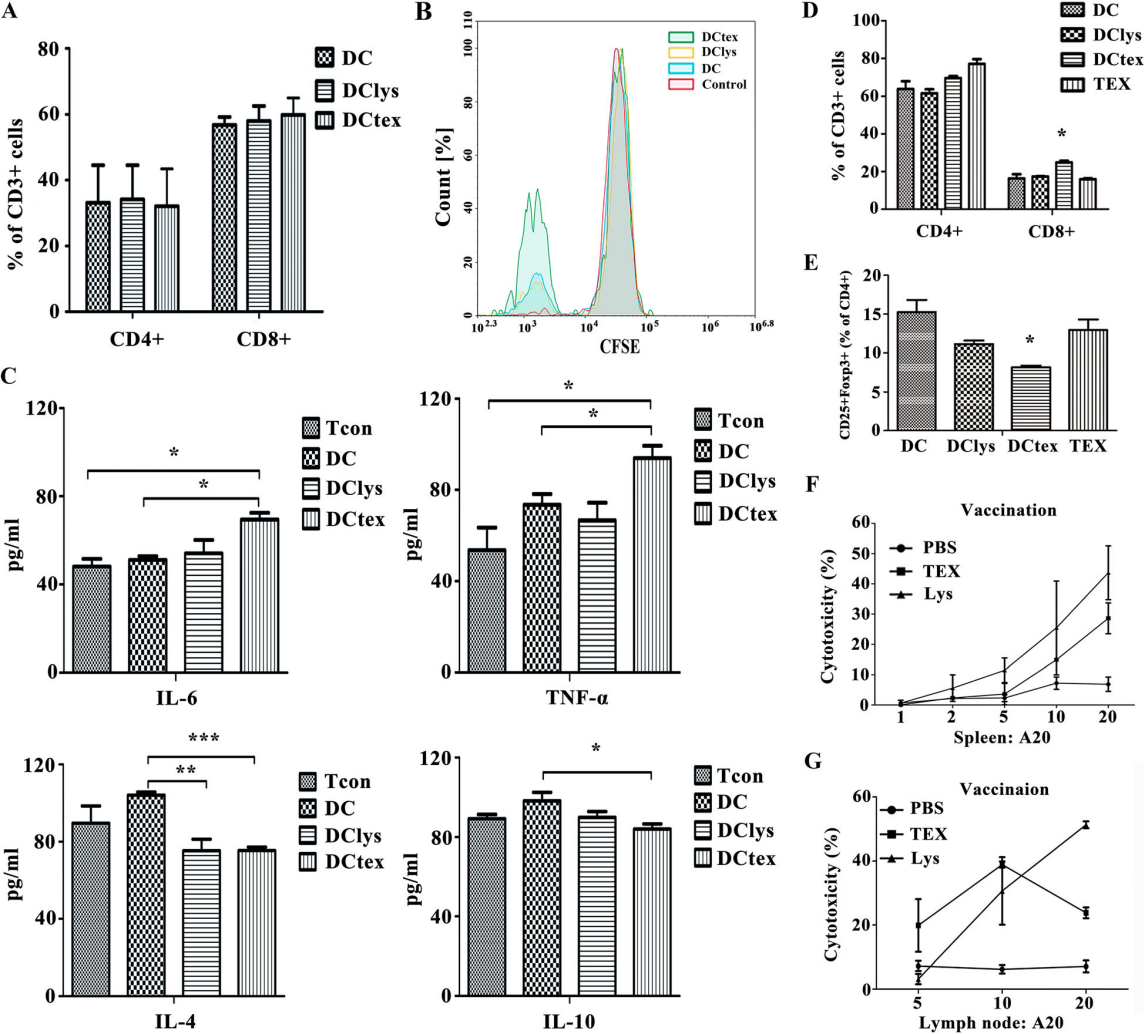
Effects of exosomes-derived from OCI-LY3 on T cell proliferation and antitumor activity. **a** Human PBMCs were co-cultured with DCs, OCI-LY3-pulsed DCs (DCtex) and cell lysates of OCI-LY3-pulsed DCs (DClys) for 4 days. Then CD3+, CD4+ and CD8+ T cells were detected by flow cytometric analysis. **b** T cell proliferation was assessed using a Carboxyfluorescein diacetate succinimidyl ester (CFSE) assay, followed by flow cytometry analysis. **c** After co-culture for 4 days, concentrations of IL-6, TNF-α, and immunosuppressive cytokines including IL-4 and IL-10 in the supernatants were measured by Cytometric Bead Array Human Th1/Th2 Cytokine Kit (two-tailed t test, *P < 0.05; **P < 0.01; ***P < 0.001). **d** BALB/C mice were immunized with DCs, A20 EXOs-pulsed DCs, or A20 lysates-pulsed DCs (DClys) for three times intravenous injection at weekly interval before challenged with A20 cells at one week after the last immunization. Then PBMNCs were obtained from the mice, and flow cytometry analysis was performed for the detection of CD3, CD4, and CD8 positive cells. **e** CD4+ CD25 + FoxP3 + Treg cells in PBMCs from the mice treated with DCtex, DClys, DC or PBS, respectively. (two-tailed t test, *P < 0.05, *n* = 6). **f** Spleen lymphocytes and (**g**) lymph node lymphocytes were harvested from the mice immunized with A20 TEXs, A20 cell lysates or PBS for 7 days, and then were co-cultured with A20 cells at different E: T (effector: target) ratios. Cytolysis efficiency of lymphocytes was evaluated by a lactate dehydrogenase-releasing cytotoxicity assay

To assess the effect of lymphoma TEXs on the ability of DCs to stimulate T cells responses, human lymphocytes separated from healthy donors were co-cultured with DCs pulsed with OCI-LY3 EXOs (DCtex) or lysates of OCI-LY3 (DClys), respectively. FACS analysis showed that an increase in the number of CD8+ T cells compared with the DClys and DCs groups, although without any significant statistical difference (Fig. 4a). Furthermore, CFSE assay demonstrated a higher stimulatory capacity of DCtex at activating naïve T cell clonal expansion compared with DClys, DCs, and PBS control (Fig. 4b). Next, we investigated the immune response induced by DCtex through determining the levels of cytokine in the supernatants. Using an CBA assay, we found that the levels of proinflammatory cytokine IL-6 and TNF-α in DCtex group were higher than in control groups (IL-6, DCtex vs. DCs *P* = 0.02; TNF-α, DCtex vs. DCs *P* = 0.046), while immunosuppressive cytokine IL-4 and IL-10 that produced by T help 2 cells was inhibited by DCtex (IL-4, DCtex vs. DCs *P* < 0.001; IL-10, DCtex vs. DCs *P* < 0.05) (Fig. 4c). However, there was no significant difference in the levels of IL-2 and IFN-α among these groups (data not shown). To investigate the capability of DCtex to trigger antitumor immune response in vivo, TEXs were obtained from mouse lymphoma cell line A20, and then A20 TEXs were co-cultured with mouse DC cell line (D2SC/1). Next, BALB/C mice were injected intravenously with DCtex, DClys, DCs, and TEXs, respectively, and then received a subsequent challenge with A20 cells by subcutaneous implantation. Examination of T lymphocytes in serum from the mice received DCtex showed a significant increase in the number of CD8+ T cells (Fig. 4d) and a decrease in the number of CD4 + CD25 + FoxP3+ Treg cells (Fig. 4e), compared with DClys, DCs, and TEXs group. We further assessed whether TEXs can induce antitumor activity. As shown in Fig. 4f, the spleen lymphocytes obtained from mice treated with TEXs showed stronger killing activities against A20 lymphoma cells at different E:T (effector: target) ratio than those of tumor lysates and PBS control. Similar results were obtained with the lymphocytes of lymph nodes (Fig. 4g).

### B cell lymphoma-derived exosomes upregulated inhibitory receptors PD-1, CTLA-4 and BTLA, and induced apoptosis of T cells through activation of Fas/FasL pathway

**Fig. 5:**
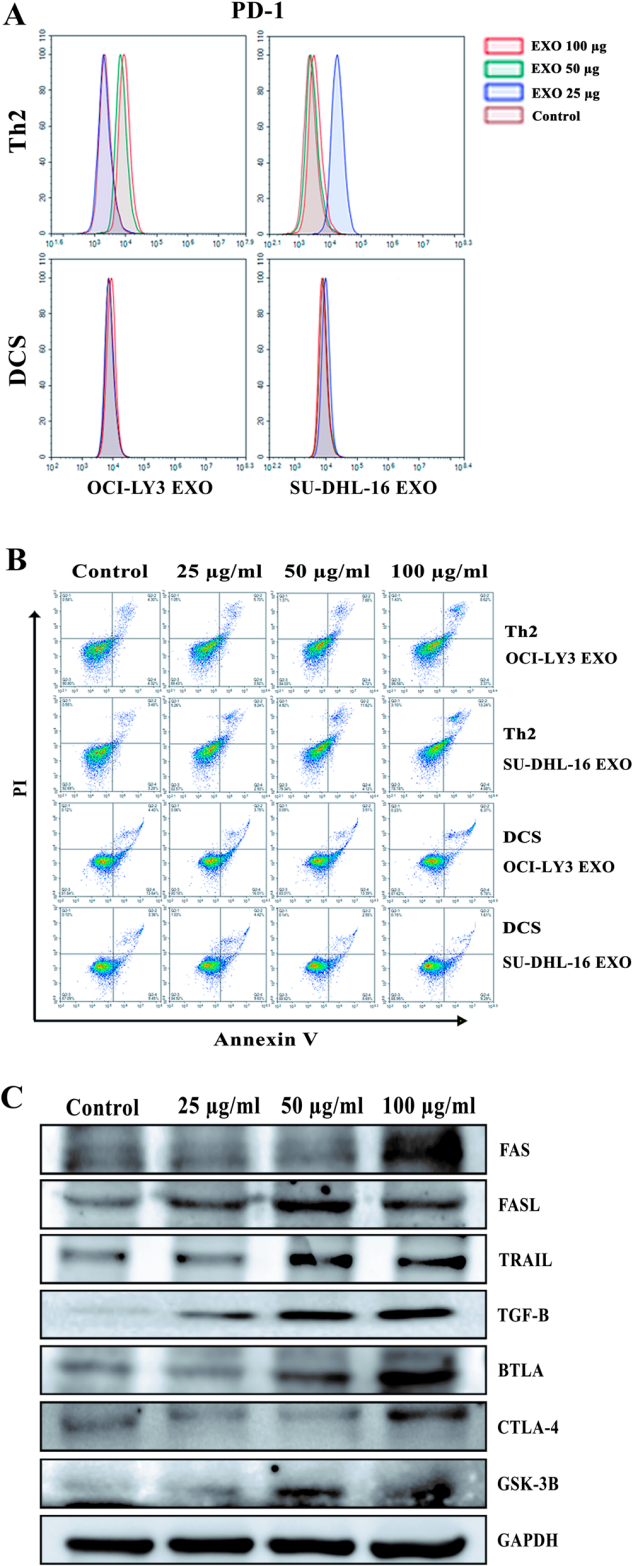
Inhibitory effect of lymphoma EXOs on T cell and DCs. **a** T cell line Th2 and DC cell line DCS were treated with EXOs-derived from OCI-LY3 and SU-DHL-16 for 24 h, and then stained with anti-PD-1 antibody, followed by flow cytometric analysis. **b** After treatment with DLBCL EXOs at the indicated doses, apoptosis of Th2 cells and DCS cells were assessed by an Annexin V/PI-staining method and flow cytometry analysis. **c** Western blotting analysis of Th2 cells treated with increasing concentrations of OCI-LY3 EXOs for 24 h

Next, we further compared the effect of lymphoma TEXs on DC cell line (DCS) and T-cell line (Th2). The cell lines were treated with different doses of OCI-LY3 EXOs and SU-DHL-16 TEXs, respectively, and expression of PD-1, an immunoinhibitory receptor, was analyzed by FACS. As shown in Fig. 5a, an induced PD-1 expression by TEXs was observed in Th2 cells, but not in DCS cells. Furthermore, treatment of Th2and DCS cells with OCI-LY3 EXOs or SU-DHL-16 TEXs for 24 h resulted in apoptosis of Th2 cells, but also not in DCS cells (Fig. 5b). To explore the mechanisms underlying TEXs-induced apoptosis and immunosuppression, apoptotic signaling molecules and some key immune inhibitory molecules were analyzed by Western-blot technique. The results suggested that the ability of TEXs to induce T-cell apoptosis was due to the upregulation of Fas, FasL, and TRAIL (Fig. 5c). Of particular note, exposures to OCI-LY3 EXOs also elicited a marked increases not only in TGFβ protein, the factor known to promote conversion of conventional T-cells into Treg cells, but also inhibitory molecules BTLA and CTLA-4, in a dose-dependent fashion (Fig. 5c).

### B cell lymphoma-derived exosomes facilitate invasion of HUVEC and human normal fibroblasts

**Fig. 6:**
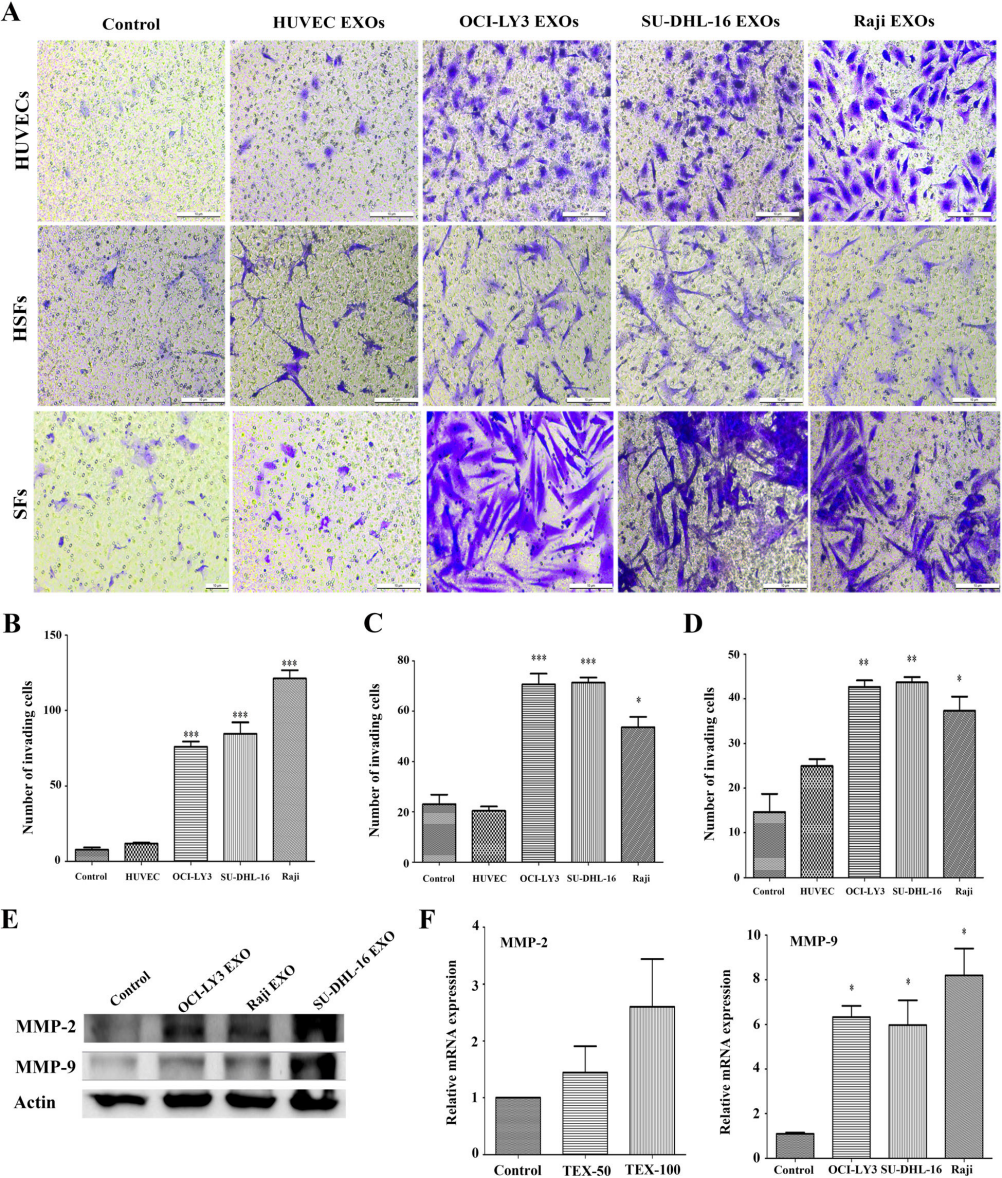
Exosomes of B cell lymphoma induce the invasion of human normal cells. **a** HUVECs, the human skin firoblasts (HSFs) and primary gastric related fibroblasts (SFs) from healthy donors were stimulated with serum-free DMEM medium containing 100 μg/ml OCI-LY3 EXOs, SU-DHL-16 EXOs, and Raji EXOs, respectively. EXOs of HUVECs and PBS were used as controls. Cells were overlaid onto Matrigel-coated Transwell inserts, and then were continue cultured for 24 h. The inserts were fixed in 4% formaldehyde and stained with 0.1% crystal violet. Slides were photographed and the invading cells counted using ImageJ software. **b** Number of the invading cells in HUVECs group. **c** Number of the invading cells in HSPs group. **d** Number of the invading cells in SFs group. Scale bar = 10 μm. Data representative of 3 independent experiments. (two-tailed t test, *P < 0.05; **P < 0.01; ***P < 0.001). **e** Western blots of MMP2 and MMP9 expression in HUVECs stimulated with 100 μg/ml of lymphoma-derived EXOs for 24 h. **f** The mRNA expression levels of MMP2 and MMP9 in HUVECs treated with lymphoma EXOs were detected by quantitative real-time PCR. (two-tailed t test, *P < 0.05)

It was well known that neoplastic B cells co-evolve with normal cells to produce a pro-survival, immunosuppressive microenvironment in lymph node and bone marrow [32]. We then asked whether HUVECs and human normal fibroblasts including the skin fibroblasts (HSFs) and primary gastric related fibroblasts (SFs) can be affected by lymphoma TEXs. Figure 6a-d shows that treatment with EXOs-derived from OCI-LY3, SU-DHL-16 or Raji cells resulted in a clear and significant increase in invasion of three kind of human normal cell lines; however, there is no effects were observed when using EXOs-obtained from HUVECs. Since MMPs, especially MMP2 and MMP9 play important role in tumor invasion and in metastatic process, and are activated in TME in DLBCL [33, 34], we next evaluated the stimulatory effect of lymphoma TEXs on MMP2 and MMP9. As seen in Fig. 6e, MMP2 and MMP9 expression levels in HUVECs were significantly upregulated after incubation with TEXs. Consistent with this data, significant increases in mRNA levels of MMP2 and MMP9 were observed in the cells treated with TEXs compared with untreated cells (Fig. 6f), suggesting that B cell lymphoma-derived EXOs play a critical role in triggering invasion of human normal cells via the activation of MMP molecules.

### B cell lymphoma-derived exosomes not only enhance cell proliferation, migration and angiogenesis of stromal cells, but also promote tumor growth in vivo

**Fig. 7:**
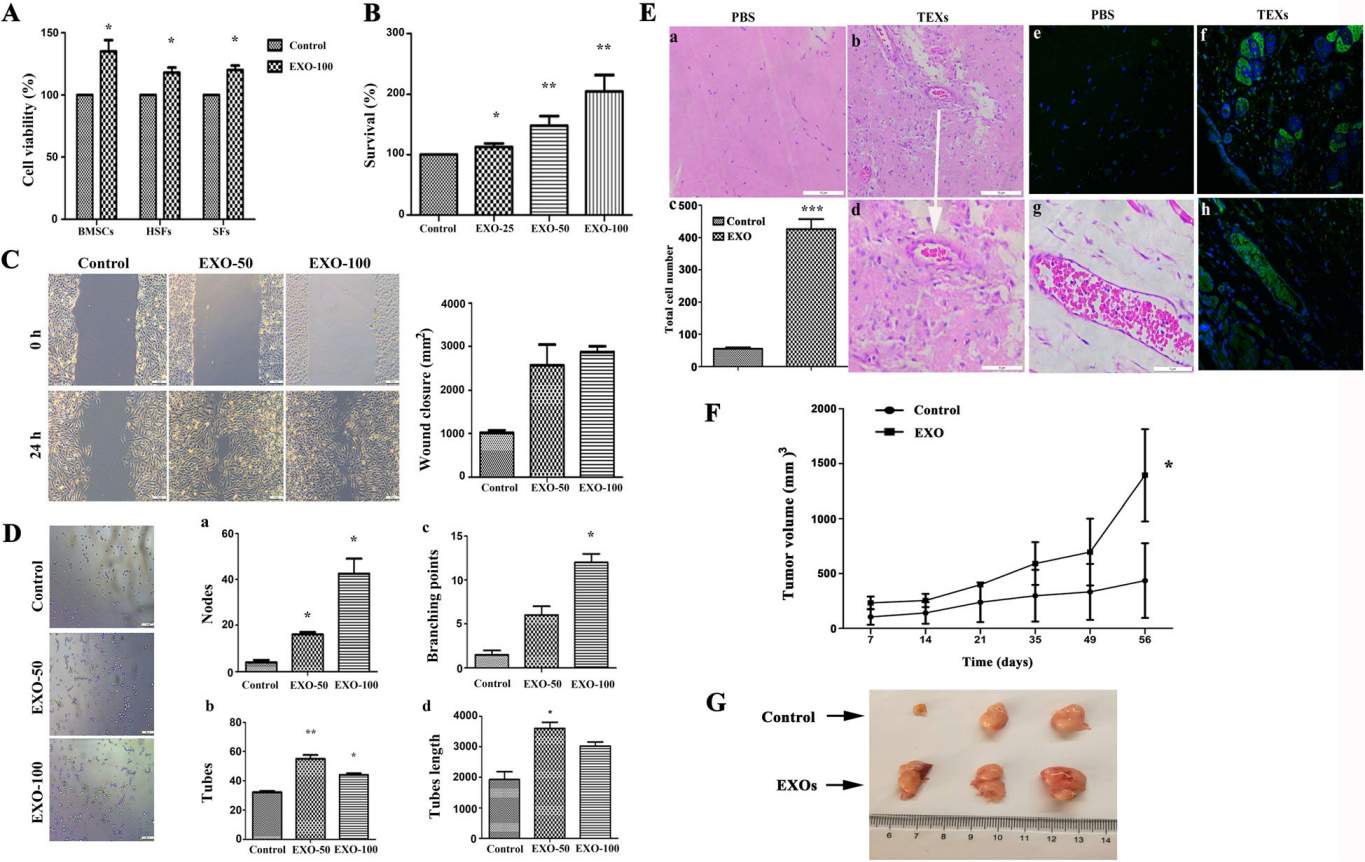
DLBCL exosomes promote cell proliferation, migration and angiogenesis in vitro. **a** Proliferation of stromal cells after 96 h of incubation with increasing concentrations of OCI-LY3 EXOs were evaluated by an MTT assay. Data are representative of three independent experiments. (two-tailed t test, *P < 0.05). **b** DLBCL cells were co-incubated with supernatants of BMSCs that were treated with or without increasing concentrations of OCI-LY3 EXOs for 24 h. After 5 days incubation, cell viability was assessed by an CCK8 assay. Data are reported as the percentage relative to control (*n* = 3). *P < 0.05; **P < 0.01. **c** (Left) Microscopy images of wound healing assay showing closure of the scratch when HUVECs were cultured in presence or absence of OCI-LY3 EXOs (50 or 100 μg/mL) for 24 h. Scale bar = 10 μm. (Right) Wound closure (μm^2^) was quantified using ImageJ software. (*n* = 3). **d** (Left) HUVECs were exposed to 50 or 100 μg/mL OCI-LY3 EXOs (50 and 100 μg/mL) or PBS (Control) for 30 min, and then seeded on Matrigel for 3 h. Scale bar = 10 μm. Quantification of several parameters of the tube formation assay using Image J (*n* = 5). (a) Nodes; (b) Tubes; (c) Branching points; (d) Tubes length. *P < 0.05, **P < 0.01. **e** Matrigel plug assay performed by subcutaneous injection of Matrigel mixed with or without OCI-LY3 EXOs (100 μg/ml) in 3 mice. Representative of H & E staining of matrigel plug: (a) PBS (200 ×); (b) OCI-LY3 EXOs (200 ×); (c) Quantitation of the total number of cells within the matrigel plug; (d) OCI-LY3 EXOs (400 ×). Representative of immunofluorescence of CD31 and DAPI co-staining of the matrigel plug: (e) PBS (200 ×; (f) OCI-LY3 EXOs (200 ×); (g) OCI-LY3 EXOs (H & E staining, 400 ×); (h) OCI-LY3 EXO (400 ×). **f** PBS (control) or 100 μg OCI-LY3 EXOs were mixed with OCI-LY3 cells and subcutaneously injected into the NOD/SCID mice, and then xenograft tumors were monitored. (*N* = 3) *P < 0.05. **g** Representative images of subcutaneous tumors removed from NOD/SCID mice at 56 days

We further assessed the proliferation of bone marrow stromal cells, fibroblasts as well as SFs after treatment with OCI-LY3 EXOs. Results show that lymphoma TEXs can enhance proliferation of these recipient cells (Fig. 7a and b) Furthermore, scratch closure test results demonstrated that the migration of HUVECs increased at 24 h in the presence of OCI-LY3 EXOs as compared to control group (Fig. 7c). Angiogenesis results from the production of proangiogenic factors by both cancer and non-malignant cells in the TEM. As it associates with poor outcome in DLBCL [35], we thus study whether DLBCL TEXs have an impact on angiogenesis by a matrigel tube formation assay. As shown in Fig. 7d, TEXs facilitate formation of capillary-like structures in HUVECs in a dose-dependent manner. To evaluate the effects of DLBCL TEXs on cell migration and proangiogenic function in vivo*,* the matrigel plugs containing 100 μg of OCI-LY3 EXOs or equivalent amount of PBS were injected subcutaneously into the abdomen of NOD/SCID mice. After 14 days, the implanted plugs were separated. H&E staining showed that TEXs treatment resulted in significantly increased number of infiltrating host cells compared to the PBS control, and induced the formation of blood vessel (Fig. 7e). Finally, we investigated the impact of OCI-LY3 EXOs on tumor growth in vivo by subcutaneously injecting OCI-LY3 cells with or without TEXs into NOD/SCID mice. Coinjection of OCI-LY3 cells with TEXs seems to increase the growth rate of implanted tumors (Fig. 7f and g).

### Comparative studies of proteomic characterization of DLBCL-derived exosomes

**Fig. 8:**
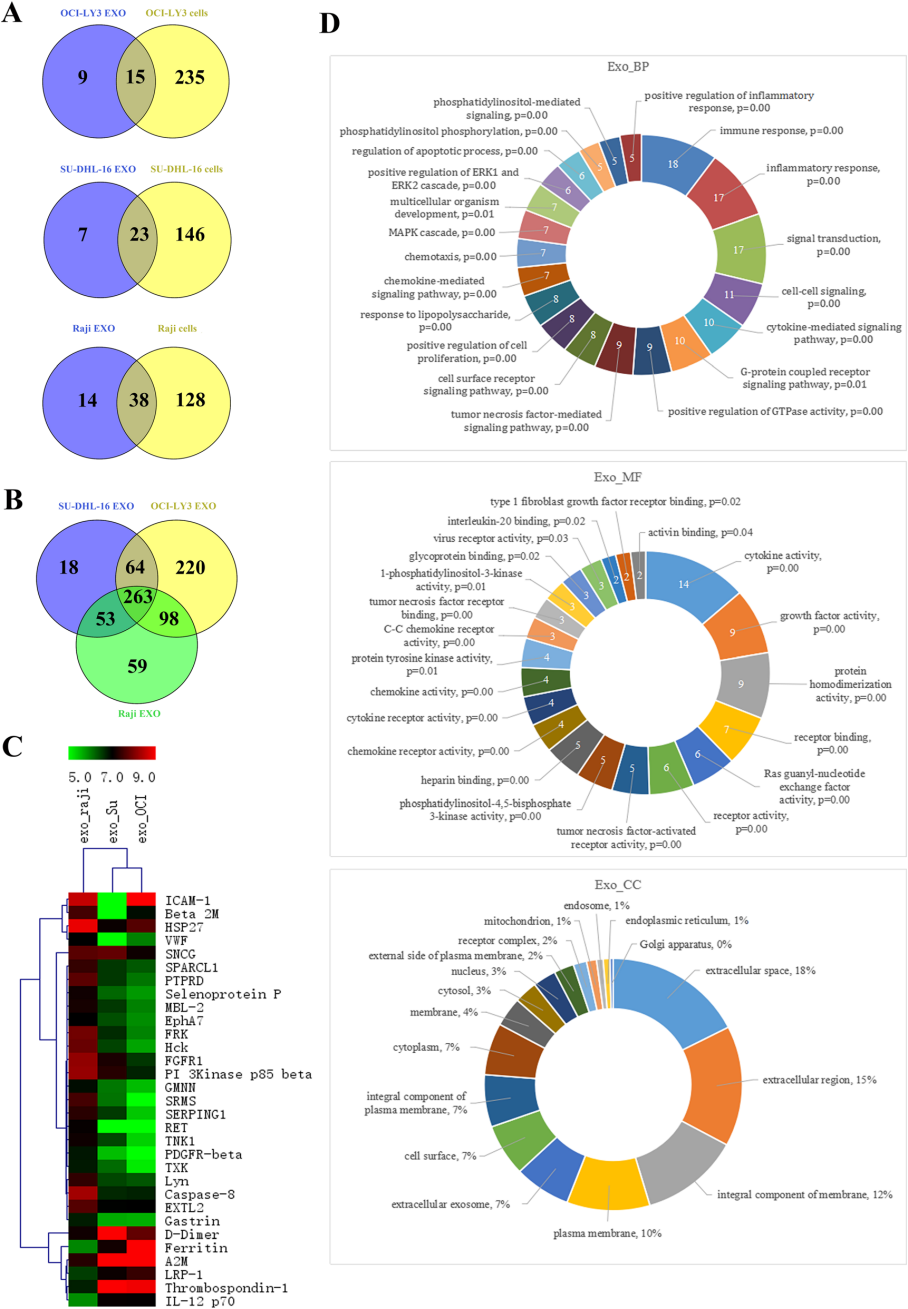
Proteomics analysis of exosomes isolated from B-lymphoma cell lines. **a** Venn diagram of proteins identified in OCI-LY3, SU-DHL-16, and Raji cells, and its corresponding EXOs. **b** Venn diagram of proteins identified in OCI-LY3 EXOs, SU-DHL-16 EXOs, and Raji EXOs. **c** A heatmap showing enrichment of exosomal proteins in EXOs derived from Raji, SU-DHL-16, and OCI-LY3 cells, respectively. **d** The indentified proteins in both OCI-LY3 EXOs and SU-DHL-16 EXOs were analyzed by GO (a) BP, biological process; (b) MF, molecular function; and (c) CC, cellular component annotation

We finally investigated the protein expressions of OCI-LY3, SU-DHL-16 and Raji cells, as well as its corresponding TEXs using an antibody array that can detect 1000 human proteins among which 508 proteins had been previously observed in EXOs (www.exocarta.org). As shown in Fig. 8a, 660 proteins were commonly identified in OCI-LY3TEXs and OCI-LY3 cells while 14 proteins were unique in OCI-LY3 EXOs. Five hundred fourteen proteins were detected in both SU-DHL-16 cells and its EXOs while 27 proteins were unique in SU-DHL-16 EXOs. Together, these data indicated that some proteins in lymphoma TEXs may be enriched in the process of EXOs development or obtained from the extracellular microenvironment. Next, we compared different proteins identified from DLBCL EXOs and Burkitt lymphoma EXOs (Fig. 8b). A majority of the proteins (263) were identified in common between the DLBCL and Burkitt lymphoma EXOs while 18, 220 and 59 proteins were unique to SU-DHL-16 EXOs, OCI-LY3 EXOs and Raji EXOs, respectively; however, hierarchical clustering of gene expression data shows a similar expression pattern between SU-DHL-16 EXOs and OCI-LY3 EXOs. Whereas, Raji EXOs have a different protein expression pattern (Fig. 8c). These data suggest TEXs cargo of DLBCL is different from Burkitt lymphoma. Using GO analysis in DAVID Bioinformatics Resources 6.7. (http://david.abcc.ncifcrf.gov/), uniquely detected proteins in bothSU-DHL-16 EXOs and OCI-LY3 EXOs were categorized by biological process (BP), cellular component (CC) and molecular function (MF) (Fig. 8d). These data will help us better understand the biologic characteristics of DLBCL TEXs.

## Discussion

Cancer cell-derived EXOs, also known as TEXs, have the ability to promote a favorable microenvironment that supports tumor growth, and to induce formation of new vessels and contribute to the metabolic reprogramming of cancer cells providing means for their sustained proliferation [36, 37]. Koch R, et al. [38] demonstrated that DLBCL possesses a self-organized infrastructure comprising side population (SP) and non-SP cells and that this transition between clonogenic states is regulated by EXO-mediated Wnt signaling. In addition, TEXs also have the ability to stimulate extracellular matrix remodeling, cancer cell migration and invasion [28, 36]. Importantly, TEXs play a crucial role in the escape of the cancer to immune surveillance [11–13]. However, relatively few studies have evaluated characterization of lymphoma cell-derived EXOs and its role in DLBCL. Our study now shows that EXOs derived from both OCI-LY3 and SU-DHL-16 cells are membrane-bound vesicles heterogeneous in size, with a mean diameter of 173.8 nm and 167.9 nm, respectively. Like other EXOs, they carry the exosomal markers CD81 and CD63, and endosome-associated proteins such TSG101 and ALIX. Importantly, using flow cytometry analysis, we observed the presence of surface markers related to malignant B-cell lineages such as CD19 and CD20, which is consistent with previous studies that show that different B-cell surface proteins (CD19, CD20, CD24, CD37 and HLA-DR) are expressed on EXOs from B-cell lymphoma cell lines [7, 39]. The presence of MHC and co-stimulatory molecules in EXOs is immunogenic; however, some kind of TEXs does not carry these molecules [40]. Here we show that SU-DHL-16 EXOs displayed expression of CD80 and CD86 similar to, but in less extent to its parental cells. Whereas, OCI-LY3 EXOs did not show any CD40, CD80, CD86, and CD83 molecules, indicating the heterogeneity of lymphoma TEXs.

TEXs contain TAAs that can be efficiently taken up by DCs, thereby eliciting specific anticancer immunity [29, 30]. It was reported that T-cell lymphoma TEXs contain tumor antigens CD24 and HSP-70 [17]. Our data show that DLBCL TEXs carry not only HSP-70 but also c-Myc, Bcl-2, Mcl-1, xIAP and Bcl-xL molecules. Furthermore, among the cargoes identified in DLBCL TEXs, molecules involved in phosphatidylinositol, ERK, MAPK, chemokine, cell surface receptor, and G-protein, etc., signaling pathway are related with cell proliferation, apoptosis resistance, and antitumor immunity. EXOs-derived from CLL and MCL cells have been demonstrated to enter and deliver their content such as miRNA and proteins to malignant B-cells and normal cells including mesenchymal stem cells and endotherlial cells [26, 28, 41]. Our results provide evidences for the uptake of DLBCL TEXs by DCs and B lymphoma cells. Given the fact that TEXs carry both TAAs and immunosuppressive mediators [29], we investigated the effect of TEXs-derived from DLBCL cells on DCs, T-cells, and TME. Using flow cytometric analyses and Western blotting, we demonstrated that OCI-LY3 EXOs are either able to up-regulate expression of PD-1 or to induce the apoptosis of Th2 cells. However, these effects were not observed in DCs. In agreement with previous studies [29, 42], our data show a dose-dependent increased expression of Fas, FasL, and TRAIL in Th2 cells treated with TEXs, which may contribute to induction of apoptosis. In addition to immunosuppressive effects of DLBCL TEXs, we also demonstrated that the TEXs play an important role in not only enhancing cell proliferation, invasion, and migration of stromal cells HUVEC and human fibroblasts, and angiogenesis, but also promoting tumor growth in vivo. Taken together, targeting lymphoma TEXs by silencing or inhibition of TEXs production may be a promising therapeutic approach.

DCs can be exploited for vaccination against cancer, which aims at stimulating tumor-specific immune responses to prevent, treat or eradicate tumors. However, therapeutic efficacy frequently remains below expectation [43]. TEXs contain TAAs, even or tumor-specific antigens that can be transferred to DCs, thereby enhancing anticancer immune responses [7, 44]. In T-cell lymphoma, TEXs bearing the marker of malignancy CD24 and HSP-70elicited specific immune responses and immune memory that allowed the rejection of subsequent tumor challenges [17]. Similar results have been reported in leukemia models [45, 46]. In this regard, our results show that TEXs did not induce apoptosis of DCs. Moreover, DCs pulsed with DLBCL TEXs have a higher stimulatory capacity in both inducing expansion of T-cells and inhibiting secretion of immunosuppressive cytokine by T helper 2 cells. The lymphocytes from mice treated with TEXs demonstrated a specific anti-lymphoma activity. Collectively, our results suggest that DLBCL TEXs can provide a source of TAAs to enhance a DC-based immunotherapeutic effect.

## Conclusions

**Fig. 9:**
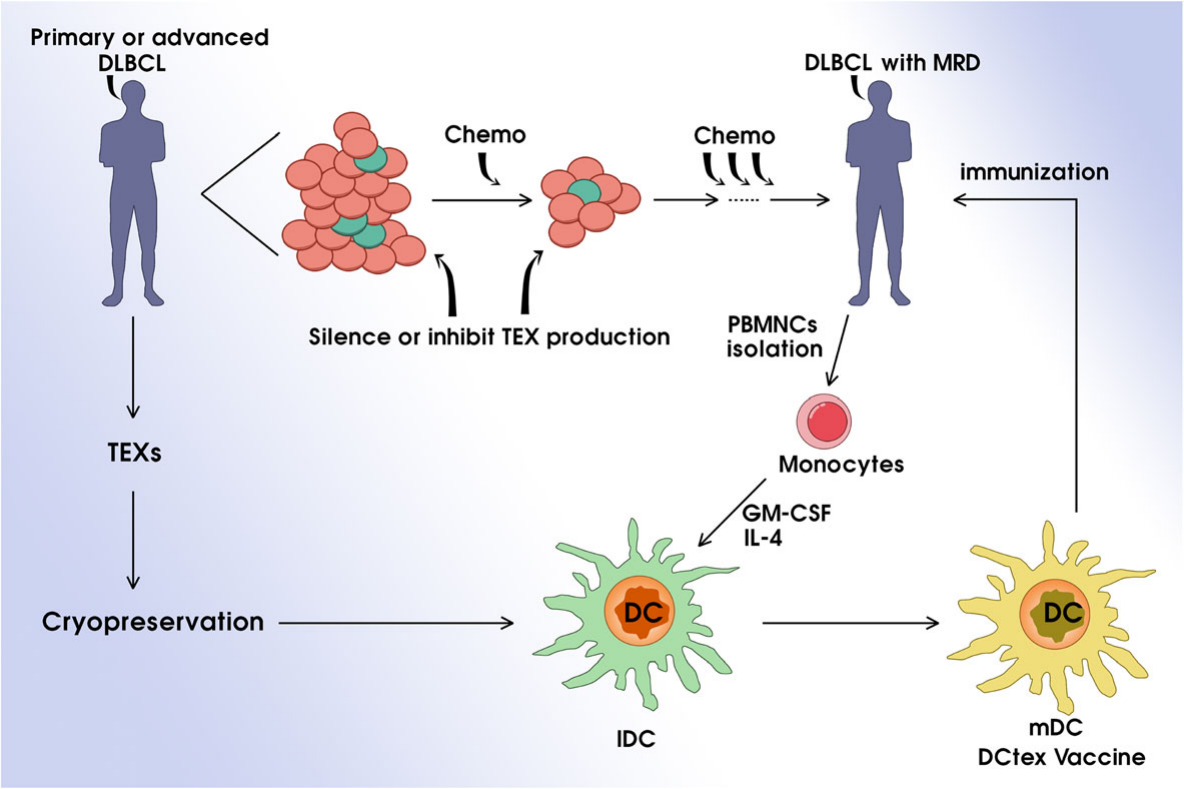
Schematic representation of the therapy targeting exosomes-derived from DLBCL. TEXs, Exosomes derived from tumor cells; Chemo, Chemotherapy; MRD, Minimal residual disease; PBMNCs, Human peripheral blood mononuclear cells; DC, Dendritic cell; IDC, Immature dendritic cell; mDC, Mature dendritic cell; DCtex vaccine, TEXs- pulsed DC vaccine

In conclusion, our findings indicated that DLBCL TEXs mainly act as an immunosuppressive mediator and stimulate cell proliferation, invasion, and migration of stromal cells HUVEC and human fibroblasts, and angiogenesis; therefore, promote tumor growth in vivo, suggesting it is necessary to target TEXs by silencing or inhibition of TEXs production. On other hand, DLBCL TEXs can also mediate and enhance DC-based antitumor immunity. Together, our data provide the framework for novel therapies targeting TEXs in DLBCL (Fig. 9).

## Additional file

Additional file 1: **Figure S1**. Immunophenotypic of B-lymphoma cell lines. The OCI-LY3, SU-DHL-16, and Raji cells were stained with a panel of antibodies, followed by flow cytometry analysis. (JPG 5093 kb)
