# Supplementary figures and images for: Dual effect of DLBCL-derived EXOs in lymphoma to improve DC vaccine efficacy in vitro while favor tumorgenesis in vivo
Source: J Exp Clin Cancer Res. 2018 Aug 13;37:190. doi: 10.1186/s13046-018-0863-7 (PMC6090784; doi:10.1186/s13046-018-0863-7)

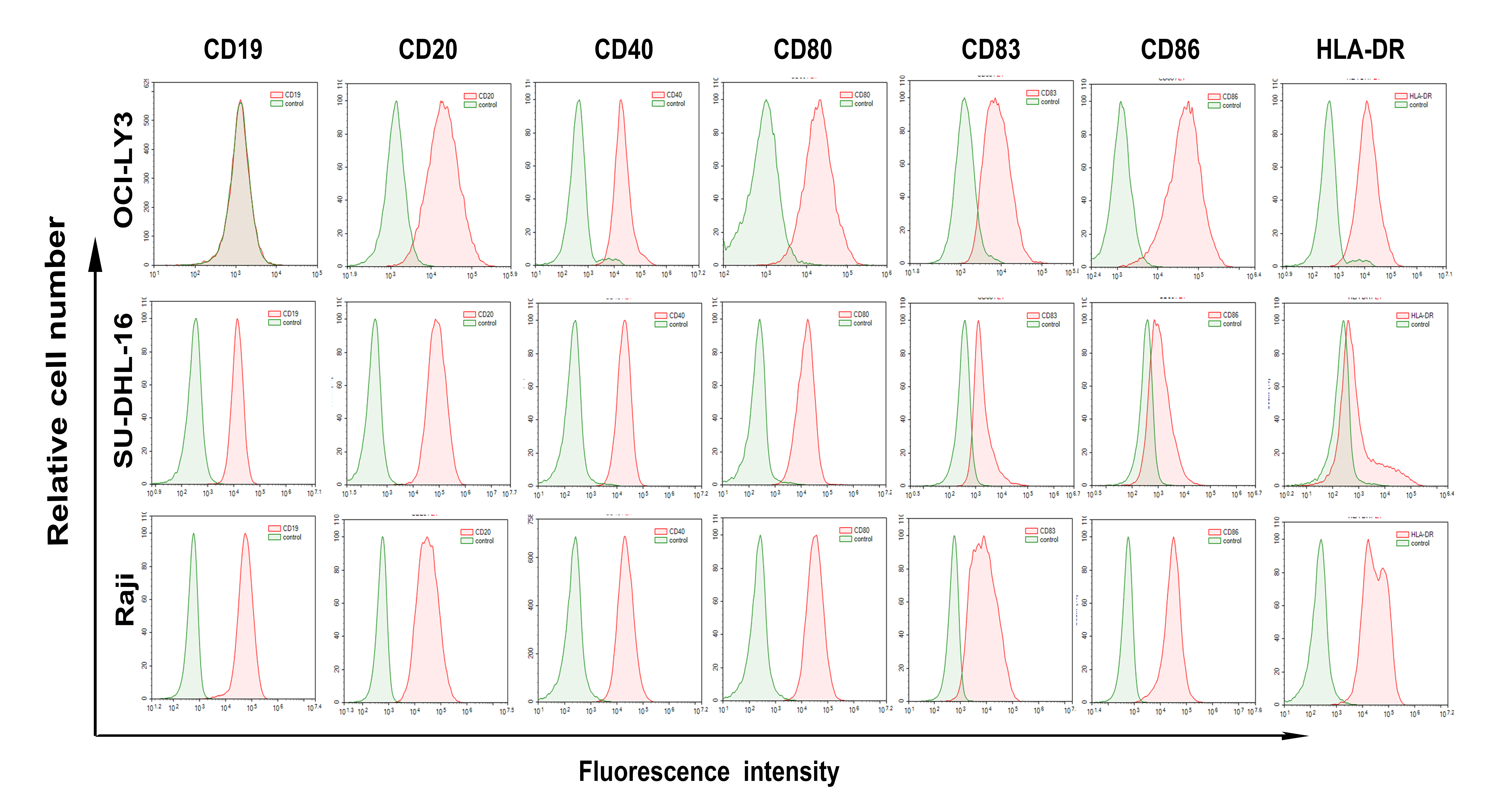

Supplement: Supplementary file 1 — Figure S1. Immunophenotypic of B-lymphoma cell lines. The OCI-LY3, SU-DHL-16, and Raji cells were stained with a panel of antibodies, followed by flow cytometry analysis. (JPG 5093 kb) [file 13046_2018_863_MOESM1_ESM.jpg]
